# Monitoring coronary blood flow by laser speckle contrast imaging after myocardial ischaemia reperfusion injury in adult and aged mice

**DOI:** 10.3389/fcvm.2024.1358472

**Published:** 2024-02-12

**Authors:** Juma El-Awaisi, Dean P. J. Kavanagh, Neena Kalia

**Affiliations:** Microcirculation Research Group, Institute of Cardiovascular Sciences, College of Medical and Dental Sciences, University of Birmingham, Birmingham, United Kingdom

**Keywords:** laser speckle contrast imaging LSCI, myocardial infarction, coronary microcirculation, ageing, ischaemia-reperfusion injury, interleukin-36

## Abstract

**Introduction:**

Investigating coronary microvascular perfusion responses after myocardial infarction (MI) would aid in the development of flow preserving therapies. Laser speckle contrast imaging (LSCI) is a powerful tool used for real-time, non-contact, full-field imaging of blood flow in various tissues/organs. However, its use in the beating heart has been limited due to motion artifacts.

**Methods:**

In this paper, we report the novel use of LSCI, combined with custom speckle analysis software (SpAn), to visualise and quantitate changes in ventricular perfusion in adult and aged mice undergoing ischaemia-reperfusion (IR) injury. The therapeutic benefit of inhibiting the actions of the pro-inflammatory cytokine interleukin-36 (IL-36) was also investigated using an IL-36 receptor antagonist (IL-36Ra).

**Results:**

Imaging from uncovered and covered regions of the left ventricle demonstrated that whilst part of the LSCI flux signal was derived from beating motion, a significant contributor to the flux signal came from ventricular microcirculatory blood flow. We show that a biphasic flux profile corresponding to diastolic and systolic phases of the cardiac cycle can be detected without mathematically processing the total flux data to denoise motion artifacts. Furthermore, perfusion responses to ischaemia and postischaemia were strong, reproducible and could easily be detected without the need to subtract motion-related flux signals. LSCI also identified significantly poorer ventricular perfusion in injured aged mice following IR injury which markedly improved with IL-36Ra.

**Discussion:**

We therefore propose that LSCI of the heart is possible despite motion artifacts and may facilitate future investigations into the role of the coronary microcirculation in cardiovascular diseases and development of novel therapies.

## Introduction

The primary goal in treating myocardial infarction (MI) is to rapidly restore blood flow in occluded epicardial arteries. However, successful revascularisation procedures can result in sub-optimal myocardial perfusion within coronary microvessels as a consequence of ischaemia-reperfusion (IR) injury (1). These microcirculatory perturbations are worse with age and may contribute to the poorer outcomes in older patients after an MI (2, 3). Increased clinical recognition of the importance of the coronary microcirculation has meant identifying strategies to prevent perturbations and maintain adequate perfusion within it has gained recent attention (4–6). However, clinical research into the role of the coronary microcirculation in MI has been limited primarily due to the inability of current imaging modalities to visualise the coronary microvasculature (6–8). Hence, no efficient strategy to improve microvascular flow post-MI has yet been identified. Experimental imaging of the coronary microcirculation *in vivo*, using powerful techniques such as intravital microscopy, has also remained challenging (8). This is due to the deep anatomical location of the heart in the chest wall, its low transparency, and the presence of motion from the heart and nearby lungs. Moreover, the need for expensive imaging equipment, heart stabilisation, and highly trained specialist personnel has further limited the use of this technique.

Laser speckle contrast imaging (LSCI) offers a more user-friendly, less technically challenging, and relatively inexpensive imaging modality for experimental assessment of microcirculatory perfusion (9). LSCI assesses microcirculatory flow from blood vessels near the tissue/organ surface, displaying perfusion as a pseudo-colour “heatmap”. Unlike laser Doppler flowmetry, which only acquires information from specific points, LSCI offers the main advantage of being able to make full-field, non-contact and non-scanning measurements over large surfaces in real time. Moreover, no exogenous contrast or fluorescent agents are required. The technique relies on the speckle phenomenon (9, 10). Briefly, when motionless tissue is illuminated with coherent light (visible red light or infrared), an interference pattern forms at the camera surface (speckle pattern). If the tissue contains mobile “scatterers”, typically red blood cells (RBCs), the speckle pattern fluctuates in intensity resulting in blurring. This blurring is quantified by a term called speckle contrast, which can be mathematically calculated and is inversely related to the “scatterer” or, in the case of blood vessels, as RBC velocity. Therefore, this technique can be used to visualise and quantify microvascular perfusion as arbitrary perfusion units (or flux).

Although LSCI has been used extensively in assess blood flow in many different tissues and organs (11), its application to the surgically exposed heart in experimental mouse models has been limited by the high degree of movement of this tissue. Indeed, the complex dynamics of coronary microvascular blood flow as a consequence of contraction/relaxation, as well as aortic pressure pulsation, present challenges for interpretation of beating heart laser speckle images. Due to the nature of the technique, LSCI is extremely sensitive to motion, which is ideal when wanting to detect the small-scale movements of RBCs. However, this also means that organ-related movement artifacts contribute significantly to the final calculated speckle contrast image. This paper describes the application of LSCI to the mouse beating heart and highlights its ability to identify changes in ventricular perfusion in response to myocardial IR injury, as well as dysfunctional changes in aged mice. Using intravital microscopy of the adult and aged mouse beating heart, we previously demonstrated that neutrophil adhesion was a striking and pathological feature of myocardial IR injury. Interestingly, these events took place primarily within coronary capillaries leading to a subsequent decrease in functional capillary density (12, 13). However, inhibiting the actions of interleukin-36 (IL-36), a novel pro-inflammatory cytokine, decreased neutrophil recruitment and improved the presence of perfused capillaries, suggesting this therapy was vasculoprotective through its actions on the coronary microcirculation. Hence, we used LSCI to determine whether it could also detect improvements in overall ventricular perfusion after pharmacological inhibition of IL-36.

## Materials and methods

### Myocardial IR injury

Experiments were conducted on C57BL/6 adult (2–4 months) or aged (18–19 months) female mice in accordance with the Animals (Scientific Procedures) Act of 1986 under a project licence (P552D4447) issued by the UK Home Office. Anaesthesia was induced and maintained by an intraperitoneal administration of ketamine hydrochloride (100 mg/kg) and medetomidine hydrochloride (100 mg/kg), and appropriate depth was confirmed by checking the pedal reflex every 15 min. Mice were intubated and ventilated with medical oxygen via a MiniVent rodent ventilator (stroke volume: 220 μl, respiratory rate: 130 pm; Biochrom Ltd/Harvard Apparatus). For some studies, recombinant mouse IL-36Ra (15 μg/mouse; Novus Biologicals) was injected via a cannulated carotid artery at 10 min pre-reperfusion and 60 min post-reperfusion (12, 13). Myocardial IR injury was performed as described previously (12–14). Briefly, a left thoracotomy was performed to allow access to the heart and the left anterior descending (LAD) artery was suture ligated for 45 min. The occlusion was then removed and reperfusion was allowed to proceed for 2.5 h. At the end of experiments, mice were killed by cervical dislocation.

### Laser speckle contrast imaging and image processing using in-house software, SpAn

LSCI was utilised to quantitate myocardial microcirculatory blood flow in the left ventricle (LV). An LSCI device (moorFLPI-2; Moor Instruments, UK) was positioned approximately 30 cm above the heart that had been kept exposed with the aid of a chest retractor. A near-infrared laser diode, which emitted a low powered laser after passing through a diffuser, was used to illuminate the heart. Prior to making comparisons between experimental groups, we checked whether the flux signal was not purely or predominantly attributable to an artifact of heart movement. To do this, an opaque and non-reflective piece of black card (∼1.5 mm thick) was placed on the proximal half of the exposed LV in an adult uninjured mouse. This prevented capture of a flux signal from blood flowing in the underlying muscle wall and only capture of a flux signal due to motion of the beating heart. Flux readings were then taken simultaneously from both the proximal covered and distal uncovered areas of the LV.

To assess myocardial perfusion in response to IR injury, flux data was collected during pre-ischaemia (baseline), ischaemia and every 15 min post-reperfusion. A total number of 1,400 frames were captured at each time point using the manufacturer supplied image software (mFLPI2Measure V2.0; mFLPIReview V5.0) at a frame rate of 25 Hz and using spatial processing (sliding window, time constant: 0.1 s). Using the freehand selection feature within the LSCI software, an area of interest was drawn on the LV downstream of the LAD artery ligation site for extraction of flux data. The LSCI flux data was then exported as Comma Separated Variable (CSV) data files into custom build analysis software package called *Speckle Analysis*, (SpAn; open source, available online at https://github.com/kavanagh21/SpAN). This software allowed systolic and diastolic events to be identified and collated from the LSCI flux measurements and exported into Excel. SpAn allows for the extraction of peak and trough flux values for use as surrogates for diastolic and systolic flow respectively. For comparisons between different experimental groups, only the average flux values from diastole were used to represent overall microcirculatory perfusion within the LV.

LSCI was also used to determine the impact of myocardial IR injury on the heart rate, which was calculated from the number of diastolic events (representing heart beats) over the period of a minute. Inter-beat interval, or beat-to-beat interval, refers to the time interval between individual heart beats. Heart rate variability occurs when there are changes in the time intervals between these consecutive heart beats. Therefore, the distance between each systolic cycle (between one systolic point and the next as determined by SpAn) was also calculated and averaged for each time point. The standard deviation was then calculated to identify any irregularities or variability in heart beat rhythm.

### Statistical analysis

Statistical analysis was performed using GraphPad 7.0 software. Data was firstly tested for normality prior to using an unpaired Student's *t*-test. A one-way ANOVA followed by a Sidak's *post hoc* was used to analyse multiple groups. The area under the curve (AUC) was calculated for time-course experiments and used for subsequent analysis as a summation of the entire period. AUC is a standard analysis method used for assessment of time-course experiments where there are a large number of individual time-points, providing a single integrated value representing the entirety of any given time-course. It was useful for comparing overall perfusion between different experimental groups rather than perfusion at different time-points and was calculated in GraphPad using the trapezoid rule. Data are presented as mean ± SEM, with *p *< 0.05 indicating statistical significance. LSCI results cannot be expressed in absolute perfusion values and were therefore expressed as arbitrary perfusion units and as a percentage of baseline.

## Results

### LSCI of the beating heart identifies a biphasic flux profile corresponding to diastolic and systolic phases of the cardiac cycle

**Figure 1:**
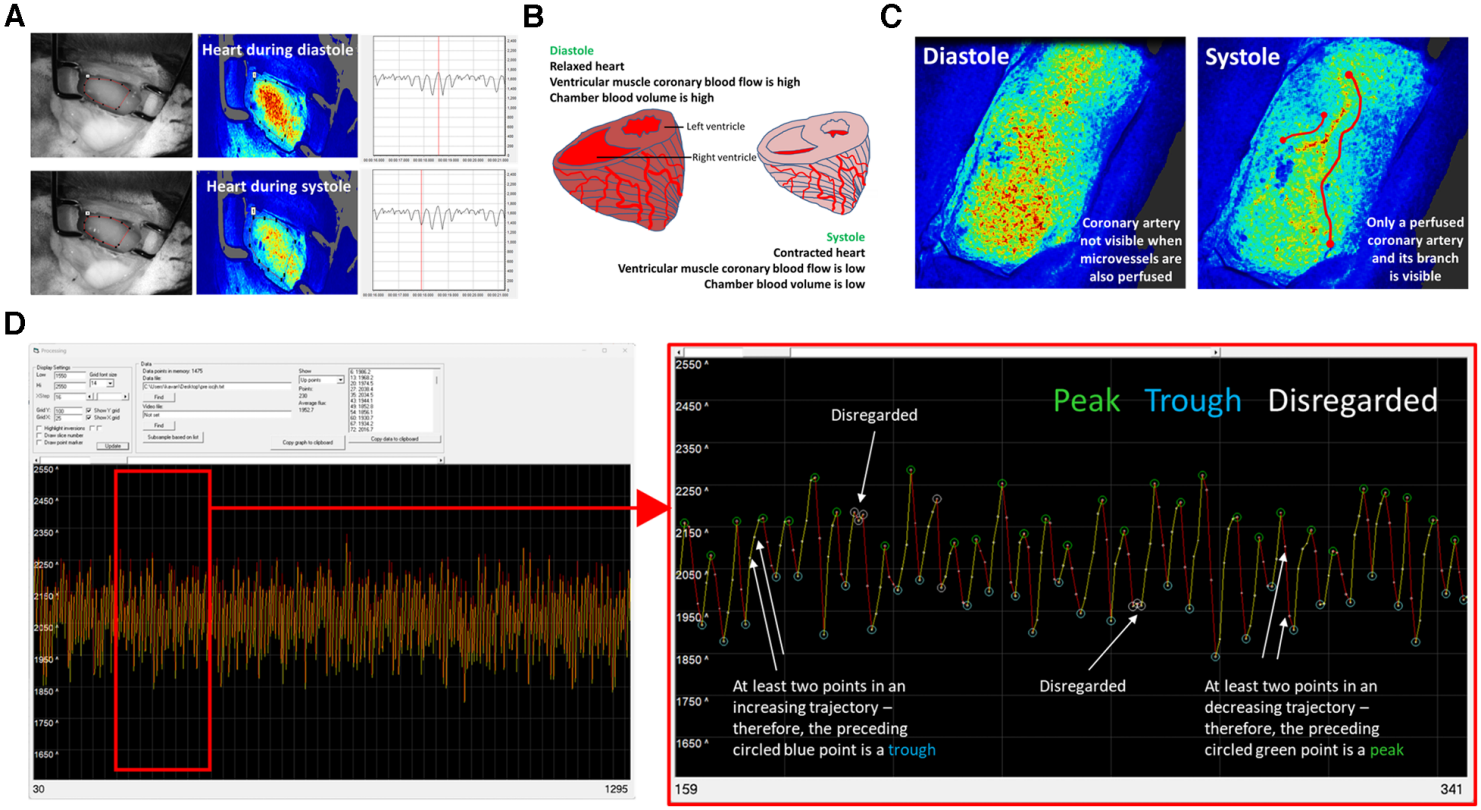
LSCI of the beating heart identifies a biphasic flux signal corresponding to diastolic and systolic phases of the cardiac cycle. (**A**) Representative LSCI heatmaps from an adult un-injured mouse heart alongside a camera photo. Diastolic and systolic phases of the cardiac cycle identified in the LSCI heatmaps as warmer and cooler colours respectively. These phases can also be clearly seen on the LSCI flux recordings. The vertical red line passes through a peak in the upper LSCI flux readout and through a trough in the lower LSCI flux readout. When correlating to the LSCI heatmap image, it is clear that these represent a time point when the heart is highly and poorly perfused respectively. (**B**) During diastole the chamber blood volume is high due to ventricular filling and myocardial blood flow is also at its highest. During systole the chamber blood flow is low due to blood ejection. Shortening of the muscle fibres compresses the microvasculature of the myocardium and thus decreases flow. LSCI only penetrates some thickness of the ventricular wall and hence recordings do not include chamber blood flow. (**C**) Whilst spatial resolution of LSCI is poor, it was sometimes possible to visualise individual coronary arteries in the heatmaps. They were only visible during systole when they appeared to remain perfused but the coronary microcirculation did not. (**D**) The averaging of data that occurs in the MoorFLPI software is carried out by the software itself. It is a simple average—the sum flux for that region of interest (ROI) divided by the total pixels in that area. SpAn software, written in-house, allows US to identify and collate the high and low points from LSCI flux data. The image on the left shows a typical SpAn flux data readout between 30 and 1,295 points, which represents approximately a 1-min LSCI recording in real-time. The image on the right is a zoomed version of the same data set between points 159–341. In order for points to be considered as high and low peaks, the two points prior and after them must have the correct trajectory i.e., high peaks must be followed by two decreasing flux points. If not, these peaks are disregarded (white circles). These high (green circles) and low (blue circles) inflection points were collected and used for analysis. The data presented in this paper analyses the high points or flux during diastole.

In each cardiac cycle, there are two points at which the heart is still. This is during peak diastole (when myocardial perfusion is high) and during peak systole (when myocardial perfusion is low due to mechanical constriction of the vessels during cardiac contraction). At these points, the LSCI flux signal is much less likely to be influenced by cardiac contraction and more driven by blood flow. These two phases of the cardiac cycle were characteristically and clearly identified in the LSCI heatmaps as warmer colours during diastole and cooler colours during systole. These combined events also presented as an oscillating flux profile in the LSCI flux readouts, with peaks observed during diastole and troughs during systole (Figures 1A–D).

SpAn software was used to identify and collate the high and low points from the LSCI flux data. In order for SpAn to consider a peak as a high point and a trough as a low point, the software had to detect at least two prior concurrent movements within the appropriate trajectory (either upward or downward steps), i.e., a low point that was preceded by two decreasing points and followed by at least two increasing points. If this was achieved, SpAn marked these high and low points for extraction for further analysis (labelled in green and blue circles respectively on outputs). If this was not achieved, the software omitted these points from analysis (marked in white circles (Figure 1C).

### LSCI flux signals from the beating heart are a sum of myocardial perfusion and LV motion

**Figure 2:**
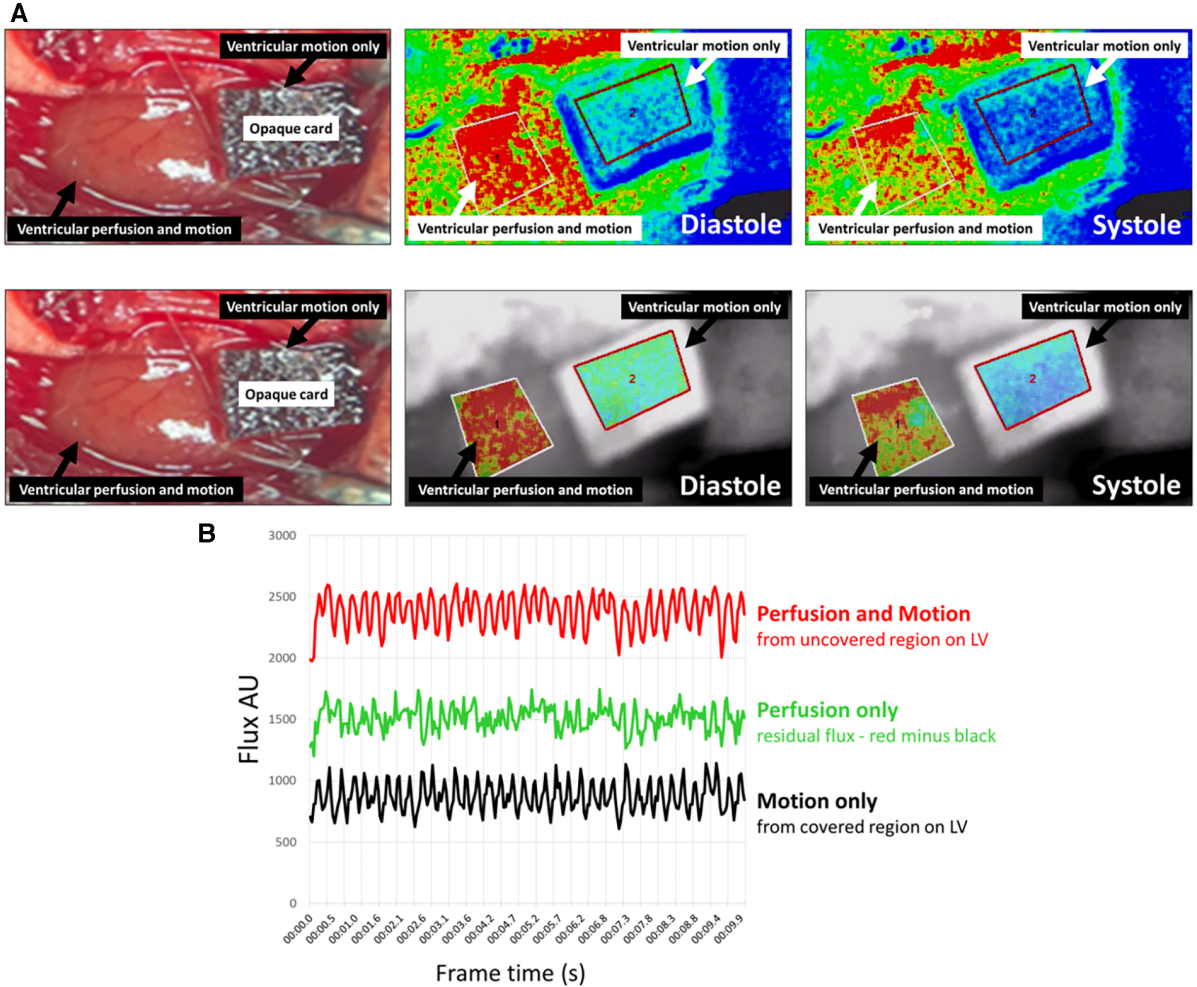
LSCI flux signals from the beating heart were a sum of myocardial perfusion and motion of the left ventricle (LV). (**A**) Representative LSCI heatmaps from an adult un-injured mouse heart. LSCI could generate heatmaps of the whole field of view (**upper panels**) or just a demarked area of interest (**lower panels**). An opaque piece of card was placed proximally on the exposed LV preventing capture of a flux signal from blood flowing in the underlying muscle wall and only capture of a flux signal due to motion of the beating heart. The heatmap in the uncovered “perfusion and motion” area alternated between being warm red colours during diastole and cooler red/green colours during systole. The heatmap in the covered “motion only” region was cooler in colour compared to the “perfusion and motion” area and alternated between cooler green and even cooler blue colours. (**B**) LSCI flux values captured over 10-s are shown. The total flux values from an uncovered area (red line) measuring perfusion and motion were higher than those obtained from the covered area (black line) measuring motion only. Although some proportion of the total flux value was derived from LV movement, there was a significant remainder that was attributable to the blood flow (green line) events taking place in the heart—this was quantitated by subtracting the black line from the red line.

To ensure that the LSCI flux signal was not purely or predominantly due to LV motion, signals were obtained from a covered and uncovered region of the LV in an adult uninjured mouse. LSCI heatmaps generated from the uncovered “perfusion and motion” area of the LV alternated between being warm red colours during diastole when there was greater perfusion, and cooler red/green colours during systole when there was less perfusion. Since LSCI of the LV area covered by an opaque card could not detect underlying ventricular perfusion, the heatmap generated from this area was unsurprisingly cooler in colour compared to the “perfusion and motion” area. Indeed, the “motion only” heatmap in the covered area alternated between cooler green and even cooler blue colours (Figure 2A; Supplementary Videos). The LSCI flux signals mirrored the events depicted in the heatmaps with values in the uncovered region higher than those obtained from the covered region. Therefore, although some proportion of the total flux signal was derived from movement, a significant remainder was attributable to microcirculatory blood flow events taking place in the LV. When the “motion only” flux values were subtracted from the total “perfusion and motion” flux values, the resultant flux signal for “perfusion only” still demonstrated a biphasic oscillating profile due to changes in perfusion during diastole and systole (Figure 2B).

### LSCI identifies poor LV perfusion in IR injured aged hearts which improves with IL-36Ra

**Figure 3:**
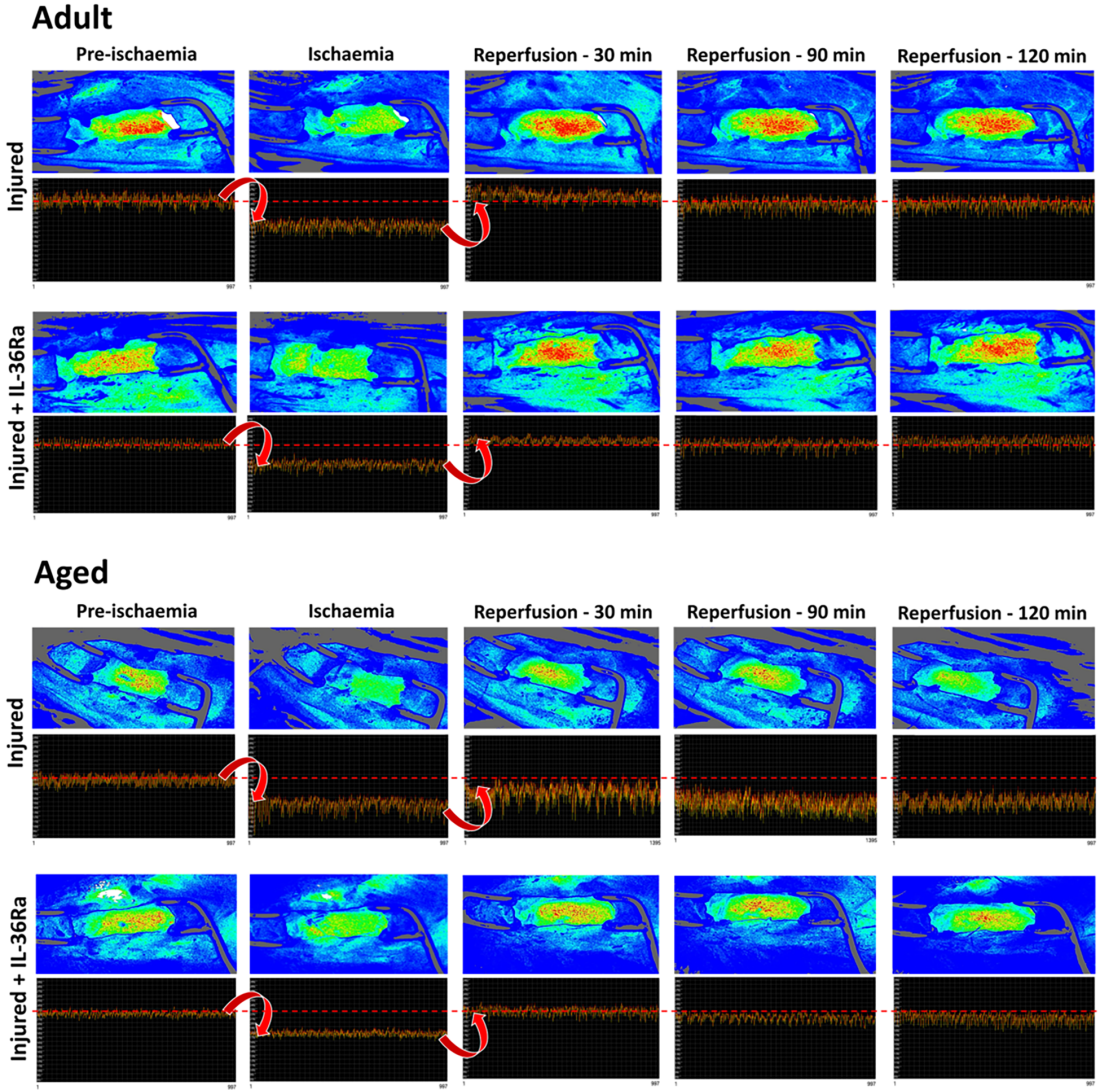
LSCI identifies poor left ventricular perfusion (LV) in IR injured aged hearts which improves with IL-36Ra. Representative LSCI heatmaps and corresponding 1-min real-time flux readouts taken from uncovered regions of the LV for untreated and IL-36Ra treated adult and aged mice undergoing myocardial IR injury. Heatmaps show warm colours under basal conditions, cooler colours during ischaemia and warmer colours again as reperfusion is initiated. Note the erratic nature of the oscillations in the flux readout for the untreated aged mouse during ischaemia and reperfusion. *n* ≤ 6/group.

Having determined that the flux signal was not purely due to LV motion, LSCI was used to assess LV perfusion in response to myocardial IR injury in untreated and IL-36Ra treated adult and aged mice (*n* = 3–7). Recordings were taken from an uncovered region of the LV downstream of the LAD artery ligation site. Heatmaps showed a rapid and striking transition to cooler colours as soon as ligation was performed in all mice. The ischaemic period was also clearly evidenced in the flux data as a marked decrease in the flux values in all mice (Figure 3). The oscillating nature of the flux signal was still present during ischaemia due to capture of LV movement. However, in untreated aged mice, the range between the mean flux at each identified up and down point pair or peak-to-peak amplitude of the oscillation was greater. Furthermore, there was also reduced uniformity of the plotted data when compared to untreated adult mice, thus appearing more “erratic”. Reperfusion restored the LSCI heatmap colour and the flux signal to basal levels in adult mice but failed to do so in aged mice. In fact, in aged hearts the flux signal continued to decrease in the reperfusion phase. Again, the oscillating flux signal continued to appear “erratic” even during reperfusion in untreated aged mice, with peak-to-peak amplitudes greater than those noted during ischaemia. IL-36Ra therapy was associated with restoration of flux to baseline values in both adult and aged mice.

**Figure 4:**
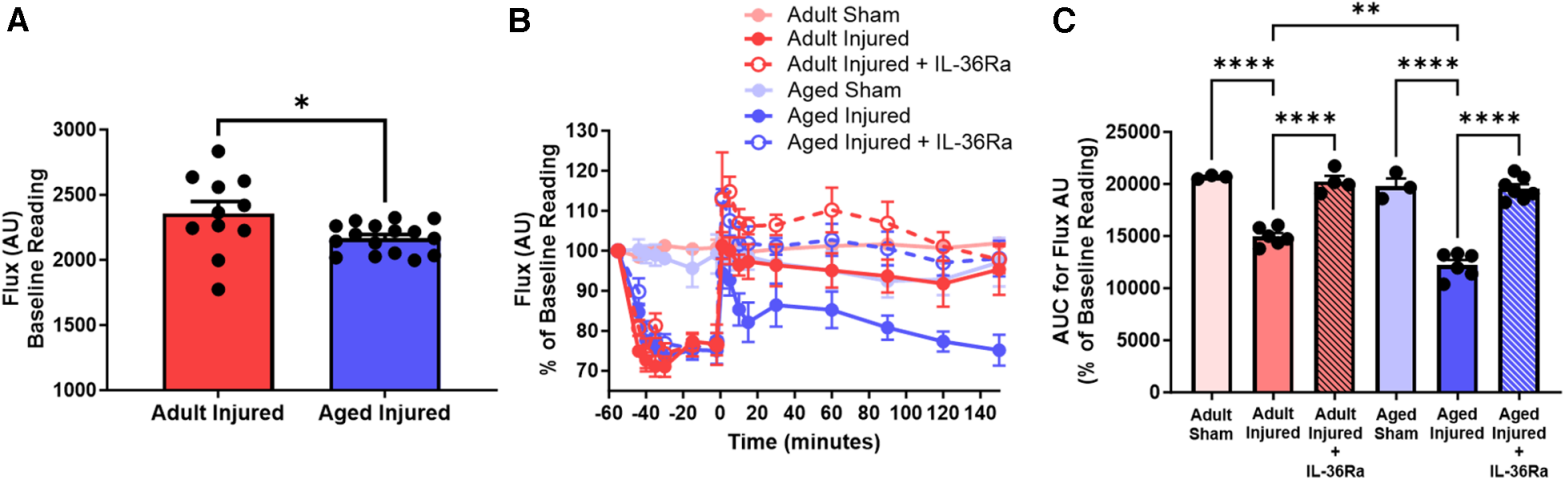
LSCI identifies poor left ventricular perfusion in IR injured aged hearts which improves with IL-36Ra. The average diastolic events between sham and untreated and IL-36Ra treated adult and aged mice, as determined by SpAn, were analysed and graphically presented for comparative purposes. (**A**) Baseline flux unit comparisons between all adult and aged mice**.** Quantitative time-course analysis of LSCI data for (**B**) flux unit and the (**C**) corresponding area under the curve (AUC) analysis for the flux unit during the reperfusion phase only. Statistical analysis was performed using an unpaired Student's *t*-test or a one-way ANOVA, followed by a Sidak's *post hoc* test. *n* = 3–7 mice/group. Data presented as mean ± SEM. *p < 0.05. **p < 0.01. ****p < 0.0001.

The average diastolic events between untreated and IL-36Ra treated adult and aged mice, as determined by SpAn, were analysed and graphically presented for comparative purposes (*n* = 3–7). Interestingly, baseline flux comparisons demonstrated that adult hearts had significantly (*p* < 0.05) higher basal flux values when compared to aged hearts (Figure 4A). As expected, ischaemia decreased the average diastolic flux signal in all mice and to a similar degree and was parallelled grossly by pallor of the LV downstream of the LAD artery ligation site. However, this never approached zero due to capture of LV movement flux signals from the uncovered region of the heart. Reperfusion, accompanied by groslly visible restoration of colour to the LV, restored the average diastolic flux signal to baseline values in adult mice but failed to do so in aged mice, mirroring the events shown in the LSCI heatmaps. Indeed, the flux signal remained below baseline values and continued to decrease throughout the period of reperfusion in aged mice. The AUC analysis for the reperfusion period showed flux was significantly (*p* < 0.01) lower in injured aged hearts when compared to injured adult hearts. IL-36Ra therapy was associated with a transient hyperaemic response in both adult and aged mice, with flux values subsequently decreasing to baseline values by the end of the imaging period. The AUC analysis for the reperfusion period demonstrated a significant (*p* < 0.0001) increase in flux in both IL-36Ra treated adult and aged treated mice when compared to their respective untreated groups. No statistically significant difference was observed between the two IL-36Ra treated groups (Figures 4B,C).

### LSCI can be used to quantitate heart rate and detect heart rhythm irregularities

**Figure 5:**
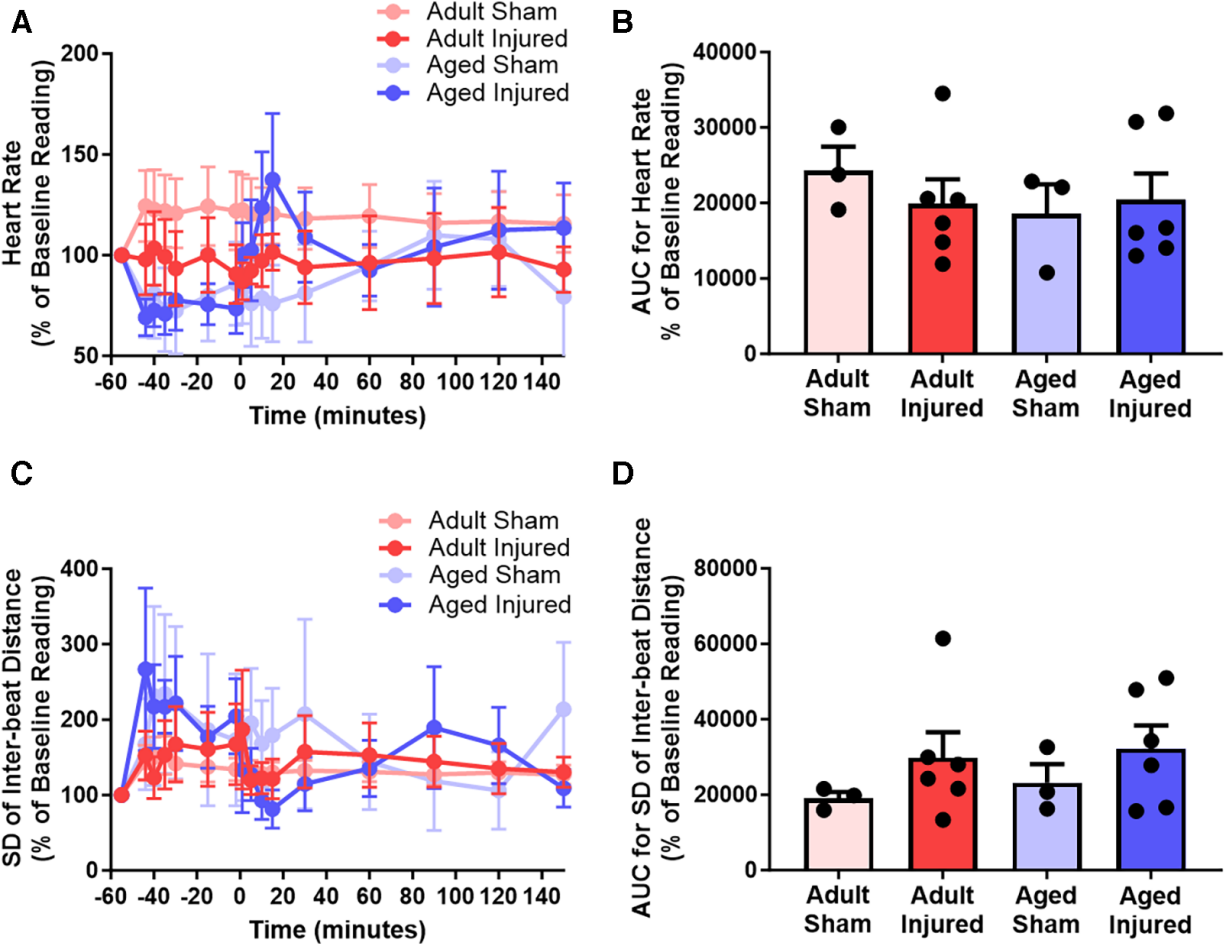
LSCI data could be used to quantitate heart rate and detect heart rhythm irregularities by assessing the inter-beat distance and subsequently the standard deviation (SD) of the inter-beat distance. (**A,B**) Quantitative time-course analysis of LSCI flux data to obtain heart rate and corresponding area under the curve (AUC). (**C,D**) Quantitative time-course analysis of LSCI flux data to obtain standard deviation of the inter-beat distance and corresponding area under the curve (AUC). Baseline capture prior to ischaemia was used as the baseline Reading. Statistical analysis was performed using an unpaired Student's *t*-test. *n* = 3–6 mice/group. Data presented as mean ± SEM.

Heart rate decreased during ischaemia and transiently increased during reperfusion in injured aged mice but remained relatively stable in injured adult mice (*n* = 3–6). However, the AUC analysis demonstrated no overall significant difference in the heart rate between adult and aged mice (Figures 5A,B). Irregularities in the rhythm of the heart beat were also detected primarily in injured aged mice during ischaemia and reperfusion. However, the AUC analysis showed no overall significant difference between adult and aged mice (Figures 5C,D).

## Discussion

Experimental use of LSCI on the beating heart *in vivo* has generally been avoided due to movement artifacts being captured alongside flux signals for perfusion. Indeed, sensitivity to movement is a major and inherent drawback of LSCI as all motion is likely to contribute to the calculated speckle contrast, regardless of whether it comes from moving RBCs or from movement of the organ being investigated. However, its application to the beating heart has gained recent interest. Plyer and colleagues applied it to the *ex vivo* perfused healthy porcine heart but primarily used it to observe coronary vasculature rather than changes in perfusion *per se*. Moreover, complicated mathematical post-processing, based on estimating displacement between acquired frames, was needed to enhance the signal-to-noise ratio of the LSCI signal (15). Liu and colleagues applied dynamic light scattering combined with LSCI to the rabbit heart. However, this was performed in a model of cardiopulmonary bypass in which cardioplegic arrest was induced to remove any contribution from cardiac motion [http://dx.doi.org/10.2139/ssrn.4454324]. The present study is the first to demonstrate that LSCI can successfully be applied to the anaesthetised mouse beating heart, without the need to restrict motion, for continuous measurement of LV perfusion after myocardial IR injury and in response to pharmaceutical intervention. Combined with our custom software and analysis process, real-time information on the changes in the coronary blood flow before, during and after myocardial ischaemia can be obtained. Furthermore, changes in analysable perfusion was identifiable in the LSCI flux signals without the need to mathematically process the data to remove motion-related artifacts. It was possible to detect the expected reduction in perfusion during ischaemia in all mice, as well as resumption of blood flow to baseline flux values immediately after the LAD artery was reperfused in adult mice. LSCI also detected a sustained and significantly poor post-ischaemic reperfusion in aged mice. These novel results demonstrate the therapeutic benefit of anti-inflammatory IL-36Ra treatment on myocardial perfusion, with transient post-occlusive hyperaemic responses also discernible.

In most tissues, blood flow peaks during ventricular systole as blood is ejected into the aorta and downstream arteries. However, blood flow through coronary vessels peaks during ventricular diastole when the muscle is relaxed. During systole, myocardial perfusion is at its lowest due to external compression of coronary microvessels by the contracting muscle (16). LSCI detected this biphasic perfusion of the myocardium as high flux peaks obtained during the diastolic phase and as low flux troughs obtained during systole. The depth of LSCI imaging is approximately 300 µm and the muscle wall thickness is 1–1.5 mm in mice. This may explain why LSCI was able to detect a biphasic response despite the fact that the most significant compressive force due to contraction is felt by microvessels in deeper subendocardial layers, with less force felt by the vessels of the epicardium (17).

A number of clinical LSCI studies have been performed in which motion artifacts have been considered (18–21). Mahe and colleagues performed one of the earliest in which they measured backscattered light signals from the forearm and also from an opaque reference area attached to the skin. They subsequently performed point-by-point subtraction of the artifact signals obtained from the opaque adhesive. The forearm underwent cuff occlusion and ischaemic and reperfusion events were investigated. Subjects were either immobile, subjected to externally produced movement to reproduce trembling or shivering patients, or were cycling (18, 19). Whilst cutaneous ischaemic and post-occlusive reactive hyperaemic events were easily identifiable in the flux signal obtained from immobile patients, they were poorly visible in moving subjects due to large amplitude motion artifacts. However, after point-by-point subtraction, or denoising of the motion signals detected from the covered forearm, the perfusion events could be optimally time-resolved even in mobile subjects. Eriksson and colleagues also compensated for tissue movement when applying LSCI to the liver microcirculation in patients undergoing liver resection. They captured events during normal breathing and apnoea when respiratory motion was absent. When the flux signal during apnoea was subtracted from the total flux signal obtained during normal beating, they demonstrated that hepatic blood flow constituted the larger part of the total signal. Interestingly, some motion signal was still obtained during apnoea which they proposed emanated from motion artifacts due to the movement of the beating heart being propagated to the liver through the diaphragm (21).

To ensure that the LSCI flux signal backscattered from the heart was not purely or predominantly due to motion, we also subtracted the flux signal obtained from a covered region of the LV from the uncovered region flux signal. Whilst motion contributed to the total measured flux signal, a larger contribution was due to LV blood flow, mimicking the results obtained by Eriksson and colleagues in the liver. Since experiments were conducted on ventilated mice, respiratory movements would have been unavoidable and would also have impacted the movement of the nearby heart. Therefore, motion artifacts due to the inherent beating of the heart and (to a much lesser extent) due to breathing would have contributed to the flux signal measured from the covered region of the ventricle. Theoretically, all our subsequent experiments should have involved point-by-point subtraction of the motion-related signal. However, myocardial infarction in the anaesthetised mouse is technically challenging, and the application and removal of an opaque card from the same region of the mouse LV when simultaneously performing LAD artery suture ligation/unligation is not practical. Therefore, whilst measurement of signals from covered and uncovered regions may be practically possible in human subjects, where large surfaces are being imaged, this is difficult to perform in the mouse heart where there is very little available ventricular surface downstream of the LAD ligature site. Indeed, the length of a mouse heart is approximately 6–10 mm, with the LV length being even shorter. Nevertheless, even without mathematically denoising for motion artifacts, we found that the impact of both ischaemia and reperfusion was discernible in the unprocessed total LSCI signal due to strong responses in LV perfusion to IR injury. For example, the lack of ventricular blood flow during ischaemia, parallelling the gross pallor of the heart, was clearly measured by a significant and rapid decrease in the total flux value.

Lending further validity to our un-denoised LSCI data is the fact that decreased perfusion in IR injured hearts of similarly aged mice was also noted in a study by Willems and colleagues, albeit they monitored coronary blood flow in excised hearts using an ultrasonic flow-probe (22). It well established that ageing is associated with a reduction in the ability of various resistance arterioles to respond to vasodilatory agonists both experimentally and in humans (23). Therefore, the ability of vasodilators and vasodilatory metabolites, known to be released and accumulate during the ischaemic phase, may not be as effective in dilating coronary arterioles in the aged heart compared to the adult heart. Reduction in nitric oxide (NO) synthesis and release, degradation of NO by reactive oxygen species, and/or increased activity of vasoconstrictive prostanoids are all potential mechanisms that could explain the poor resumption of blood flow in aged hearts (23, 24). One of the principal metabolic coronary vasodilators is adenosine which plays an important role in maintaining coronary blood flow post-ischaemia. However, it is well established that the functional responses to adenosine are decreased with age due to loss of adenosine receptors in the heart (25). Furthermore, the age-related impairment in LV perfusion may also result from an increase in the recruitment of occlusive thromboinflammatory cells within the IR injured coronary microvessels (12). This may explain the beneficial effects of the anti-inflammatory IL-36Ra on ventricular blood flow during reperfusion. Collectively, these various perturbations may all contribute to the decreased perfusion in the aged heart as observed by LSCI.

Although ascertaining whether LSCI could be applied to the mouse beating heart to quantitate myocardial perfusion was the main objective of this study, it was possible to calculate heart rate from the number of diastolic events over the period of a minute. Therefore, despite it not being the primary aim of this study, we also investigated the impact of myocardial IR injury on heart rate. Heart rate and other more broad cardiac physiological factors may influence myocardial perfusion (26). For instance, it is possible that a reduced heart rate or reduced cardiac contractility could lead to reduced LCSI flux readings due to reduced flow velocity. However, in the current study, neither IR injury alone, nor in the presence of IL-36Ra therapy, changed heart rate when compared to sham controls. We therefore believe that LCSI flux readouts in this model are driven largely by reductions and/or alterations in cardiac microvascular perfusion. Nevertheless, it would be interesting to see how LCSI performs in the context of other cardiac dysfunction models which do change heart rate such as hypertrophic cardiomyopathy, or in the presence of heart rate changing drugs such as ivabradine.

This novel *in vivo* study provides the first real-time LSCI demonstration of the rapid and detrimental impact of IR injury on ventricular perfusion in the beating heart, which was particularly heightened in aged mice. This likely contributes to the worsened outcomes observed in elderly patients after an MI. The observed benefit of an IL-36 receptor antagonist on coronary microcirculatory perfusion suggests targeting inflammation is a worthwhile pursuit. Interestingly, we also noted an erratic LSCI flux signal in response to ischaemia and reperfusion in aged mice as evidenced by greater peak-to-peak amplitudes of the flux oscillations when compared to baseline. Furthermore, the standard deviation of the inter-beat distance showed significant variation in aged mice throughout the imaging period. Hence, LSCI could potentially offer insights into the functional responses of the heart to injury in addition to information on blood flow. Importantly, our study proposes raw LSCI flux signals could be analysed to obtain robust information on coronary blood flow in response to myocardial IR injury without the need for complicated mathematical processing.

Interestingly, we noted basal changes in ventricular flow in the current study as a result of ageing and following diabetes (unpublished work). Hence, this method could be used to detect changes in coronary microcirculatory perfusion in response to other co-morbidities such as chronic kidney disease and hypertension. It may also be useful for detecting coronary microcirculatory changes in experimental models of heart failure, fibrosis, arrhythmias, in response to ischaemic pre-conditioning or for testing the impact of pharmacological agents that can modify coronary vascular tone. To conclude, we propose that LSCI of the heart is possible and may facilitate future investigations into the role of the coronary microcirculation in cardiovascular diseases and the development of novel coronary vasculoprotective therapies.

### Limitations

It is worth noting that weak coronary responses to other types of injuries or interventions may not be readily discernible using LSCI as they may be masked by stronger signals due to motion. This poses a significant limitation to the experimental application of LSCI to the beating heart *in vivo*.

## Data Availability

The original contributions presented in the study are included in the article/**Supplementary Material**, further inquiries can be directed to the corresponding author.

## Supplementary material

LSCI flux signals from the beating heart were a sum of myocardial perfusion and motion of the left ventricle (LV). Representative LSCI heatmaps from an adult un-injured mouse heart. LSCI could generate heatmaps of the whole field of view (**A**) or just a demarked area of interest (**B**). An opaque piece of card (dashed lines) was placed proximally on the exposed LV preventing capture of a flux signal from blood flowing in the underlying muscle wall and only capture of a flux signal due to motion of the beating heart. The heatmap in the uncovered “perfusion and motion” area alternated between being warm red colours during diastole and cooler red/green colours during systole. The heatmap in the covered “motion only” region was cooler in colour compared to the “perfusion and motion” area and alternated between cooler green and even cooler blue colours.
